# The Epipeptide YydF Intrinsically Triggers the Cell Envelope Stress Response of *Bacillus subtilis* and Causes Severe Membrane Perturbations

**DOI:** 10.3389/fmicb.2020.00151

**Published:** 2020-02-11

**Authors:** Philipp F. Popp, Alhosna Benjdia, Henrik Strahl, Olivier Berteau, Thorsten Mascher

**Affiliations:** ^1^Institute of Microbiology, Technische Universität (TU) Dresden, Dresden, Germany; ^2^Université Paris-Saclay, INRAE, AgroParisTech, Micalis Institute, ChemSyBio, Jouy-en-Josas, France; ^3^Center for Bacterial Cell Biology, Biosciences Institute, Newcastle University, Newcastle upon Tyne, United Kingdom

**Keywords:** *Bacillus subtilis*, antimicrobial peptides, cell envelope stress response, RiPP, epipeptide, membrane depolarization, membrane rigidity

## Abstract

The Gram-positive model organism and soil bacterium *Bacillus subtilis* naturally produces a variety of antimicrobial peptides (AMPs), including the ribosomally synthesized and post-translationally modified AMP YydF, which is encoded in the *yydFGHIJ* locus. The *yydF* gene encodes the pre-pro-peptide, which is, in a unique manner, initially modified at two amino acid positions by the radical SAM epimerase YydG. Subsequently, the membrane-anchored putative protease YydH is thought to cleave and release the mature AMP, YydF, to the environment. The AMP YydF, with two discreet epimerizations among 17 residues as sole post-translational modification, defines a novel class of ribosomally synthesized and post-translationally modified peptides (RiPPs) called epipeptides, for which the mode-of-action (MOA) is unknown. The predicted ABC transporter encoded by *yydIJ* was previously postulated as an autoimmunity determinant of *B. subtilis* against its own AMP. Here, we demonstrate that extrinsically added YydF^*^ kills *B. subtilis* cells by dissipating membrane potential via membrane permeabilization. This severe membrane perturbation is accompanied by a rapid reduction of membrane fluidity, substantiated by lipid domain formation. The epipeptide triggers a narrow and highly specific cellular response. The strong induction of *liaIH* expression, a marker for cell envelope stress in *B. subtilis*, further supports the MOA described above. A subsequent mutational study demonstrates that LiaIH—and not YydIJ—represents the most efficient resistance determinant against YydF^*^ action. Unexpectedly, none of the observed cellular effects upon YydF^*^ treatment alone are able to trigger *liaIH* expression, indicating that only the unique combination of membrane permeabilization and membrane rigidification caused by the epipetide, leads to the observed cell envelope stress response.

## Introduction

In order to establish themselves in their natural habitats, all living organisms need to defend and secure their environmental niches against rivaling species. Bacteria have developed diverse strategies to outperform competitors e.g., for limited amounts of nutrients, including the production of antibiotics (Czárán et al., 2002). One important antibiotic class that is particularly relevant for Gram-positive bacteria are antimicrobial peptides (AMPs), which usually target cell envelope integrity by a range of different mechanisms (Malanovic and Lohner, 2016). Despite the differences in their specific mode-of-action (MOA), AMPs share common structural features such as high amount of hydrophobic residues and their overall cationic charge (Guilhelmelli et al., 2013). The latter allows AMPs to bind the negatively charged surface of the bacterial cytoplasmic membrane, while the first promotes the integration and disruption of membrane homeostasis (Teixeira et al., 2012; Malanovic and Lohner, 2016). As a consequence, targeted cells suffer severe envelope damage, which inevitably results in cell death (Jordan et al., 2008).

Not surprisingly, bacteria developed different strategies to closely monitor their cell envelope and sense the presence of AMPs to ensure a rapid response in launching defensive counteractions (Staron et al., 2011; Guilhelmelli et al., 2013; Radeck et al., 2016b). The Gram-positive soil bacterium *Bacillus subtilis* harbors a complex cell envelope stress response (CESR) network orchestrated by at least four extracytoplasmic function sigma factors (ECFs) and a similar number of two-component systems (TCSs) (Jordan et al., 2008; Helmann, 2016; Radeck et al., 2016a). One such TCS, LiaRS, responds to a broad range of cell envelope stress conditions, including cell envelope perturbing agents (including AMPs), but also abiotic stresses such as heat and osmotic shock (Mascher et al., 2003, 2004). In response, it strongly induces the expression of a single operon, *liaIH*, which encodes a membrane associated protein, LiaI, and a phage-shock protein homolog LiaH (Wolf et al., 2010; Domínguez-Escobar et al., 2014; Radeck et al., 2016b).

**Figure 1:**
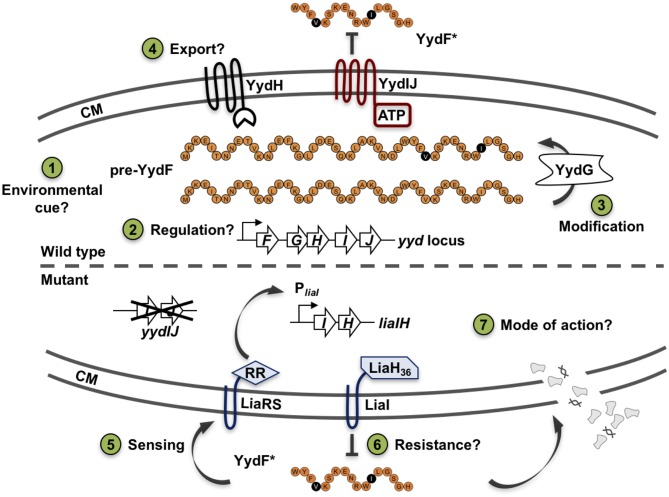
Schematic overview of the *yydFGHIJ* operon in *B. subtilis*. In the wild type (1–4), at transition from exponential to stationary phase a yet unknown cue triggers expression of the *yyd* operon (1). P_*yydF*_ is described as a constitutive σ^A^-dependent promoter, details on any regulation beyond that are still missing (2) (Butcher et al., 2007). The radical SAM epimerase YydG post-translationally modifies pre-YydF by substituting two amino acids from their L-form into their D-counterparts (3) (Benjdia et al., 2017b). Export and further processing of pre-YydF to its active from is most likely mediated by the membrane bound protease YydH (4). The ABC transporter YydIJ is postulated as potential resistance determinant (red). In a *yydIJ* knockout strain (5–7), active YydF* launches the LiaRS TCS (5) (Butcher et al., 2007). The phage-shock like protein LiaH and its anchor protein LiaI, are potentially involved in mediating resistance against YydF* (6). Detailed investigations if and how YydF* kills *B. subtilis* cells are yet to be unraveled (7). Arrows indicate activation, T-bars inhibition; CM, cytoplasmic membrane.

While the CESR of *B. subtilis* has so far been mostly studied with regard to extrinsically applied stress conditions, we recently also observed an endogenous induction of specific CESR modules in stationary phase cultures. The two cannibalism toxins SDP and SKF—AMPs that are produced to delay or even prevent the production of dormant endospores (González-Pastor et al., 2003; González-Pastor, 2011)—trigger the CESR network (Höfler et al., 2016). Additionally, a random mutagenesis study revealed genes that intrinsically activate the Lia system. Transposon insertions into the genes *yydIJ* resulted in an elevated P_*liaI*_ activity, indicative of intrinsic cell envelope stress in the absence of this postulated ABC transporter (Butcher et al., 2007). Subsequent investigations supported that the *yydFGHIJ* operon encodes a post-translationally modified peptide (RiPP) biosynthesis locus, with YydF predicted to be the epipeptide precursor (Butcher et al., 2007). Later, it was shown that YydF is a 17mer RiPP secreted in *B. subtilis* supernatant and containing, in an unexpected manner, two critical epimerizations conferring YydF with antimicrobial properties (Benjdia et al., 2017b). Hereby, a σ^A^-dependent promoter drives the expression of the *yyd* operon (Figure 1, steps 1–2). Pre-pro-YydF is initially post-translationally modified by YydG, a radical S-adenosyl-L-methionine (SAM) epimerase, by substituting two amino acids from the L-form into their D-counterparts (Benjdia et al., 2017b; Figure 1, step 3). Final processing and export of pre-YydF is presumably mediated by the membrane-bound protease YydH (Figure 1, step 4) leading to the biosynthesis of a novel class of RiPPs called epipeptides (Benjdia et al., 2017b). The co-produced ABC transporter YydIJ is postulated to provide autoimmunity against the active extracellular YydF. If this AMP acts on the cell envelope in the absence of the putative resistance mechanism *yydIJ*, an intrinsic envelope stress was observed, substantiated by LiaRS activation (Butcher et al., 2007; Figure 1, step 5). So far, neither the MOA of YydF, nor the intrinsic resistance mechanisms that *B. subtilis* can mount have been experimentally studied. Based on the data available so far, both LiaIH and YydIJ represent potential candidates (Figure 1, steps 6–7).

In this work, we address these open questions by making use of chemically synthesized mature YydF peptide (referred to as YydF^*^) extrinsically applied and thereby uncoupled form its native expression. Global transcriptome profiling of the YydF^*^ stress response results in a very narrow CESR, primarily triggering *liaIH* induction. Subsequent functional studies identify LiaIH, and not YydIJ, as the major resistance determinant against YydF^*^. Finally, we demonstrate that this AMP kills cells by depolarizing the membrane via membrane permeabilization. Moreover, YydF^*^ leads to a strong reduction in membrane fluidity, substantiated by lipid domain formation based on local differences in lipid packing. This killing mechanism enlightens the MOA of epipeptides and adds to the diversity in MOA observed for AMPs produced by Gram-positive bacteria.

## Results

### Both Chemically Synthesized and Intrinsically Produced YydF Trigger the LiaRS-Dependent Cell Envelope Stress Response

**Figure 2:**
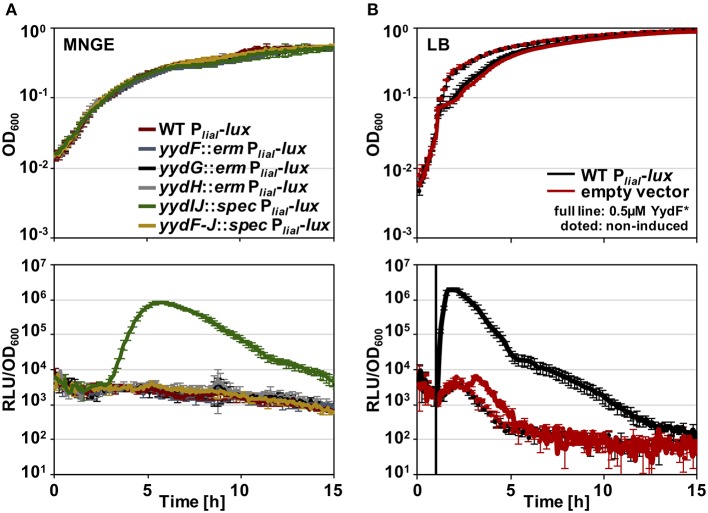
Growth curves and luciferase activity of *B. subtilis* P_*liaI*_-*lux* strains. Upper panels depict growth curves, lower panels show luminescence values normalized over optical density. **(A)** Growth in minimal media of P_*liaI*_-*lux* strains in the wild type background and individual *yyd* mutants. **(B)** Wild type P_*liaI*_-*lux* (red) and an empty vector control (blue) incubated in full medium, were challenged with extrinsic added 0.5 μM YydF* (full lines) or non-treated (dotted lines).

Before analyzing the MOA and the underlying global stress response pattern triggered by the AMP YydF, we first verified that synthetic and extrinsically applied YydF^*^ truly activates the TCS LiaRS in a comparable manner to the previously observed intrinsic induction in a *yydIJ* mutant (Butcher et al., 2007; Figure 2). In this mutant, the Lia response is indeed triggered at the transition from exponential to stationary phase (Figure 2A). At peak levels (after ~6 h of growth in MNGE medium), we observe luminescence signals of the Lia promoter that are comparable to a full induction of the system with known inducers (Mascher et al., 2004; Radeck et al., 2016b). Individual disruptions of all other genes within the *yyd* locus lead to no notable Lia promoter activity, and were comparable to the wild type setting as well as to a full knockout of the *yyd* operon (Figure 2A, lower panel). In contrast to growth in minimal (MNGE) medium, we did not observe any YydF-dependent Lia activity under nutrient-rich growth conditions (LB medium) using the same set of strains (Figure S1). This enabled us to subsequently analyze the response to extrinsically added YydF^*^ in the wild type reporter strain without interference by the intrinsic transition state induction. In LB medium, addition of 0.5 μM YydF^*^ lead to a rapid induction of the Lia promoter (Figure 2B). The response reached maximum levels within an hour post-induction and then decreased continuously over the remaining time of the experiment (Figure 2B, lower panel). At these concentrations, addition of YydF^*^ not only fully triggers the Lia response but also had a minor effect on growth, demonstrating that YydF^*^ is able to cause severe cell envelope stress to *B. subtilis* cells. Based on these initial observations, we next aimed at studying the global transcriptional response to YydF^*^-dependent cell envelope stress.

### Transcriptome Profiling of *B. subtilis* Upon YydF^*^ Treatment

**Figure 3:**
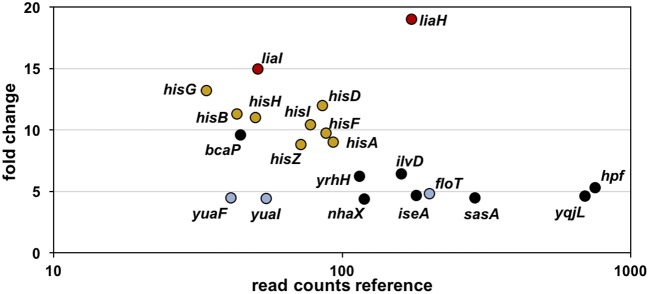
RNA-sequencing (RNA-seq) profile of *B. subtilis* upon YydF* treatment. Visualization of altered gene expression in *B. subtilis* upon 0.5 μM YydF* exposure after 10 min, compared to non-induced control samples. Fold change of elevated RNA-seq counts are plotted over read counts in the reference condition. Same colored dots highlight genes encoded within an operon. For further details, see Table 1 and Table S2.

Toward this end, we performed RNA-sequencing (RNA-seq) experiments with *B. subtilis* treated with YydF^*^ at sub-lethal concentrations (0.5 μM), which resulted in only minor growth defects. We compared the gene expression profiles between induced and non-induced samples 10 min post-induction (see methods for detail). In total, only 21 genes were differentially expressed in the presence of YydF^*^ (Figure 3, Table 1 and Table S2). Most prominent was the upregulation of the LiaRS controlled *liaIH* operon, the two target genes driven by the P_*liaI*_ promoter (Mascher et al., 2004). This observation strongly suggested the cell envelope as the main target of YydF^*^ action. Additionally, several ECF-dependent envelope stress-inducible genes (*hpf*, *yqjL, nhaX*, and *yrhH*) were also upregulated, most notably the SigW-dependent *yuaF*-*floT*-*yual* operon (Helmann, 2016). Taken together, this narrow but specific response strongly suggests that YydF^*^ causes cell envelope stress, most likely at the level of membrane-anchored steps of cell envelope biogenesis, which is the common feature of all known inducers of the Lia response. Moreover, elevated FloT levels potentially suggest alterations in membrane fluidity caused by YydF^*^ action (Bach and Bramkamp, 2013).

**Table 1:** RNA-sequencing (RNA-seq) profile of *B. subtilis* upon YydF^*^ treatment.

**Gene/Operon^**a**^**		**Regulation/** **Regulon^**a**^**	**Fold change^**b**^**	***p*-value^**b**^**	**Description^**a**^**
*liaH*	***liaIH***	LiaRS	19.1	3.2·10^−189^	Cell wall biosynthesis marker
*liaI*					
*hisG*	***hisZGDBHAFI***	YlxR	13.2	5.6·10^−55^	Amino acid biosynthesis
*hisD*					
*hisB*					
*hisH*					
*hisF*					
*hisI*					
*hisA*					
*hisZ*					
*floT*	***yuaF*****-*****floT*****-** ***yuaI***	SigW	4.5	5.6·10^−53^	Membrane fluidity marker
*yuaF*					
*yuaI*					
*bcaP*	***bcaP***	CodY	9.6	2.3·10^−49^	Amino acid transporter
*ilvD*	***ilvD***	CodY	6.5	1.6·10^−81^	Dihydroxy-acid dehydratase
*yrhH*	***yrhH***-*fatR*-*yrhJ*	SigW, SigM, SigX, SigV	6.3	3.7·10^−69^	Predicted methyltransferase
*hpf*	*yvzC*-*fliD*-*filS*-*fliT*-***hpf***	PhoP, SigB, SigH, SigD	5.3	5.9·10^−78^	General stress protein
*iseA*	***iseA***	WalR	4.7	2.1·10^−35^	Inhibits cell separation
*yqjL*	***yqjL***	SigW, SigB, SigM, SigV	4.7	6.9·10^−91^	General stress protein
*sasA*	***sasA***	SigW, SigM, SigV	4.5	2.5·10^−77^	(p)ppGpp synthetase
*nhaX*	***nhaX***	SigB, NhaX	4.4	8.7·10^−40^	General stress protein

a *Subtiwiki (Zhu and Stülke, 2018)*.

b *Highest values are depicted*.

*Bold indicates specifically induced gene*.

In addition to this specific cell envelope stress response, we observed moderate activation of genes associated with central metabolism (Zhu and Stülke, 2018). The *his* operon encoding proteins involved in histidine amino acid biosynthesis, along with *bcaP* and *ilvD*, showed increased expression. Such patterns have regularly been observed in cell envelope stress transcriptome signatures and are attributed to secondary and indirect effects of antibiotic treatment (Wecke and Mascher, 2011).

Together, this narrow and highly specific RNA-seq profile upon YydF^*^ treatment clearly points toward maintaining cell envelope integrity, with induction of *liaIH* representing the main transcriptional effect. Thus, we next investigated the potential role of the Lia system as resistance determinant against YydF^*^.

### LiaIH Mediates Resistance Against YydF^*^

**Figure 4:**
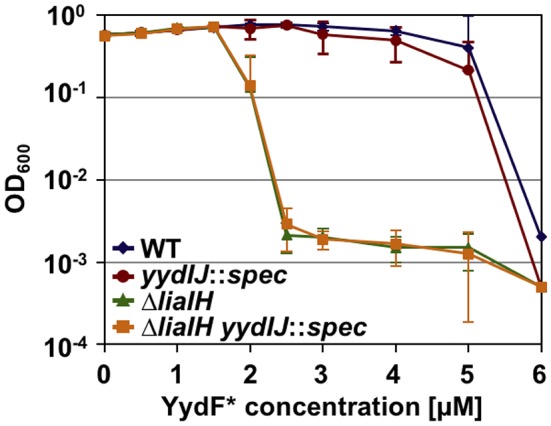
Minimum inhibitory concentration (MIC) assay of *B. subtilis* treated with YydF*. Optical density endpoint measurements after 24 h are plotted over the indicated YydF* concentrations. Next to the wild type (blue), a *yydIJ* mutant (red), a *liaIH* knockout strain (green), and the corresponding double mutant (orange) are depicted.

As a prerequisite for analyzing resistance determinant against YydF^*^, we first determined the minimum inhibitory concentration (MIC) for this AMP. *B. subtilis* cells were grown overnight, diluted in fresh LB medium and directly exposed to increasing concentrations of YydF^*^. Endpoint measurements of the optical density were taken after 24 h of incubation (Figure 4). We compared the wild type with mutant strains lacking either *liaIH* or *yydIJ*, the postulated immunity determinant encoded within the *yyd* operon, and the corresponding double mutant. Both the wild type and the *yydIJ* mutant showed MIC values of 6 μM YydF^*^ (Figure 4, blue and red line). Nevertheless, deletion of *yydIJ* showed a slight growth defect at higher YydF^*^ concentrations compared to the wild type, indicating that *yydIJ* might play a minor role in mediating resistance. In contrast, the *liaIH* deletion lead to a clear increase in YydF^*^ susceptibility, with the MIC value dropping almost three-fold (green line). Interestingly, the observed low MIC values correlate to the deemed concentrations necessary to saturate bacterial membranes (Melo et al., 2009; Melo and Castanho, 2012). Taken together, the phage-shock protein LiaH together with its membrane anchor LiaI are the major resistance determinants against YydF^*^ action. This is supported by the observation that the *liaIH*/*yydIJ* double mutant (yellow line) did not show an increase in YydF^*^ sensitivity compared to the single *liaIH* knockout (green line).

### YydF^*^ Triggers Membrane Depolarization and Causes Membrane Permeabilization

The Lia response is widely used as a sensitive marker for interference with membrane-anchored steps of cell envelope homoeostasis (Mascher et al., 2004; Rietkötter et al., 2008; Radeck et al., 2016b) and both its induction and role in protecting *B. subtilis* cells against YydF^*^ action, and the additional induction of *floT* expression pointed toward the cell envelope as the main target for YydF^*^ inhibition. Thus, we next comprehensively investigated the MOA of YydF^*^ on *B. subtilis* to (i) understand how YydF^*^ kills *B. subtilis* cells and (ii) gain deeper insight on the mechanisms required to launch a Lia response.

**Figure 5:**
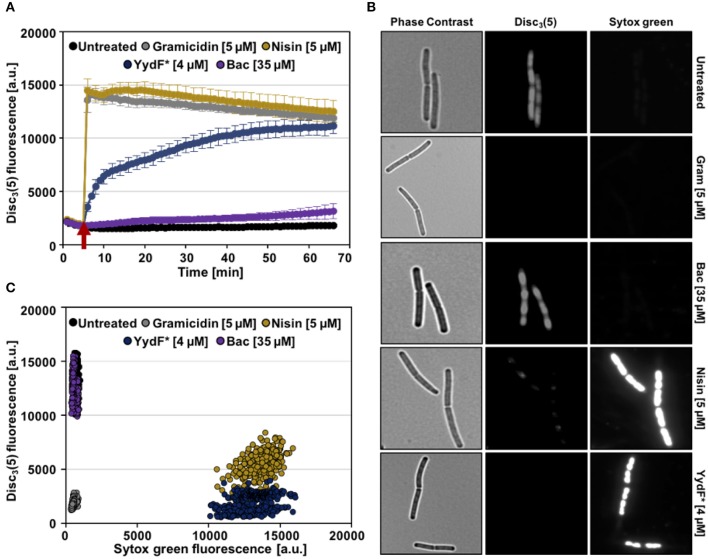
YydF* causes membrane depolarization and membrane permeabilization. **(A)** Membrane potential levels of *B. subtilis* cells upon treatment with different antimicrobial compounds were obtained using the voltage sensitive dye DiSC_3_(5). Gramicidin (5 μM, ~10xMIC) and nisin (~MIC) (gray and gold lines) were used as positive controls for rapid and complete membrane depolarization. Addition of 4 μM YydF* showed a rapid depolarization in the first 5 min after addition, followed by a gradual complete depolarization. The peptide antibiotic bacitracin at 35 μM and non-treated cells showed no membrane depolarization (purple and black lines). **(B)** Membrane potential and permeability measurements on single-cell level. Phase contrast (left panels) and fluorescence microscopy of *B. subtilis* cells stained with DiSC_3_(5) (middle panels) as well as with the membrane permeability indicator sytox Green (right panels). **(C)** Quantification of DiSC_3_(5) and sytox green fluorescence from individual cells per tested condition (~300), derived from the fluorescence microscopy experiments in **(B)**. Membrane depolarization yields in low DiSC_3_(5) fluorescence, whereas membrane permeabilization results in high sytox green signal.

First, we analyzed alterations of the membrane potential upon exposure of *B. subtilis* cells to YydF^*^ using the voltage sensitive dye DiSC_3_(5). Due to its overall hydrophobic nature, DiSC_3_(5) is able to freely diffuse across lipid bilayers. Because of its positive charge, however, the diffusion of DiSC_3_(5) is biased toward the cell interior by the inside-negative membrane potential present in well-energized cells (Strahl and Hamoen, 2010; Jahn et al., 2015; Te Winkel et al., 2016). In consequence, the slow response dye DiSC_3_(5) accumulates in cells with high membrane potential and is released into the supernatant upon dissipation of membrane potential (Miller, 2016). The membrane potential-dependent cellular accumulation of DiSC_3_(5) can be either observed directly through fluorescent microscopy, or indirectly by measuring the fluorescence quenching observed upon cytoplasmic accumulation of DiSC_3_(5) (Müller et al., 2016; Te Winkel et al., 2016; Scheinpflug et al., 2017b; Wenzel et al., 2018). As positive controls, we used the pore-forming lantibiotic nisin (Wiedemann et al., 2004) and the small cation-specific channel-forming peptide gramicidin (Kelkar and Chattopadhyay, 2007), which both rapidly depolarize *B. subtilis* membranes (Figure 5A, gray and gold lines). While exposure of *B. subtilis* to 4 μM YydF^*^, a concentration causing rapid cell death under these growth conditions (Figure S2), also led to a dissipation of the membrane potential. This reaction occurred with a much slower and rather gradual kinetic: a complete membrane depolarization was only reached after about 45 min (Figure 5A, blue line). The differences observed in membrane depolarization kinetics have been observed previously (Spindler et al., 2011), and can be explained by potential absorption of the AMP by the cell wall or alterations in membrane surface binding rate. Another study focusing on the kinetic behavior of AMPs against the Gram-negative model organism *Escherichia coli* elucidated the membrane lipid polysaccharide content as crucial for antimicrobial activity (Freire et al., 2015).

We also included bacitracin, which is the best-studied inducer of the Lia response (Azevedo et al., 1993; Ishihara et al., 2002; Radeck et al., 2016b) in the same assay. However, even upon addition of 35 μM bacitracin, a concentration below MIC however, which fully induces the Lia system, no depolarization of the membrane was observed (Radeck et al., 2013, 2016b; Figure 5A, purple line). We can therefore rule out that membrane depolarization is a requirement for Lia activation, since both bacitracin and YydF^*^ treatment causes comparable P_*liaI*_ induction despite their mechanistic differences.

We next investigated the ability of YydF^*^ to trigger membrane permeabilization. Toward this goal, we performed a fluorescence microscopic approach, which combines the voltage-sensitive dye DiSC_3_(5) with the membrane-impermeable DNA intercalating dye sytox green. In this assay, breakdown of the membrane diffusion barrier function, which can be caused either by pore formation or disruption of the continuous lipid bilayer structure, is observed as bright nucleoid stain (Roth et al., 1997; Marcellini et al., 2009; Kepplinger et al., 2018). Consequently, well-energized (that is, intact) cells exhibit high DiSC_3_(5) fluorescence, but low sytox green staining, as can be observed for the untreated cells (Figure 5B, upper row). Upon addition of nisin, which forms large cytoplasmic pores, a bright sytox green signal, accompanied by the loss of DiSC_3_(5) fluorescence was observed, indicating membrane permeabilization and the inevitably associated membrane depolarization. Treatment with 4 μM YydF^*^ for 10 min indicated a highly similar MOA: We observed a strong positive sytox green signal within cells and a simultaneous loss of DiSC_3_(5) fluorescence (Figure 5B, fifth row). As expected, cells treated with gramicidin and bacitracin showed depolarization without membrane permeabilization and a lack of membrane potential dissipation, respectively (Figure 5B, second and third row). This behavior was robust and homogenous, as demonstrated by quantifying the fluorescence signals for ~300 cells for each compound, which resulted well-distinguishable sub-populations, in agreement to their respective MOA (Figure 5C).

### YydF^*^ Reduces Membrane Fluidity and Triggers Lipid Domain Formation

**Figure 6:**
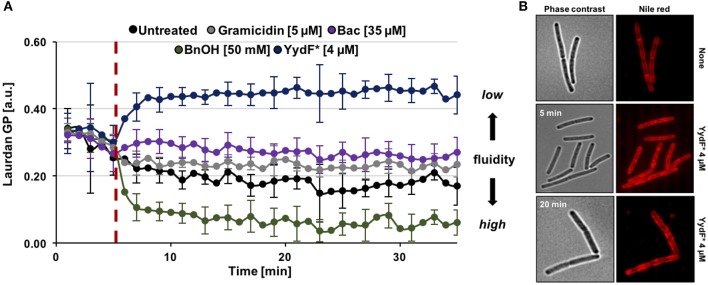
YydF* causes membrane rigidification and lipid domain formation. **(A)** Measurement of generalized polarization (GP) values using the fluidity sensitive fluorescent probe laurdan. Higher laurdan GP values correlate with rigid membrane state. Cells were incubated for 5 min to obtain basal GP levels, followed by addition of compounds (dashed red line). Benzyl alcohol was used as positive control for increased membrane fluidity, i.e., drop of GP values (green line). Non-treated, gramicidin, and bacitracin showed no effects on the fluidity state of the membrane (black, gray, and purple lines). Addition of 4 μM YydF* lead to a rigidification of *B. subtilis* cell membranes, indicated by elevated GP values (blue line). **(B)** Phase contrast and fluorescence images of *B. subtilis* cells stained with the membrane dye nile red after 5 and 20 min incubation with 4 μM YydF*, respectively.

So far, we have identified dissipation of membrane potential and membrane permeabilization as central aspects of YydF^*^ action. Under non-stressed conditions, bacteria have to maintain appropriate membrane fluidity to allow optimal activity and lateral diffusion of membrane proteins, while simultaneously ensuring sufficiently low membrane permeability (Zhang and Rock, 2008). We therefore investigated if YydF^*^ treatment also interferes with membrane fluidity in *B. subtilis*. Again, a fluorescence-based assay was performed, using the fluidity-sensitive dye laurdan. This probe alters its emission spectrum as a direct consequence of the amount of water molecules available between lipid head groups, thus providing a measure for lipid packing density (Parasassi and Gratton, 1995; Scheinpflug et al., 2017a). Indeed, a clear increase in laurdan generalized polarization (GP) values was observed upon addition of 4 μM YydF^*^ (Figure 6A, blue line). Elevated GP values were obtained within 3 min of induction, suggesting membrane rigidification as a direct consequence of YydF^*^ treatment. This rapid loss in membrane fluidity, could explain the observed delayed depolarization kinetics upon YydF^*^ treatment. As a control, the known membrane fluidizer benzyl alcohol was incorporated in the assay, which indeed lead to the opposite effect (Scheinpflug et al., 2017a; Figure 6A, green line). No changes in membrane fluidity were observed upon treatment with gramicidin and bacitracin (Figure 6A, gray, purple, and black lines). We therefore could rule out that altering membrane fluidity was a necessary feature for launching a Lia response in *B. subtilis*, since both YydF^*^ and bacitracin induce *liaIH* expression, but only YydF^*^ causes membrane rigidification.

Finally, we investigated whether YydF^*^ causes altered membrane lipid packing by using a nile red dye-based assay (Strahl et al., 2014; Scheinpflug et al., 2017b). Indeed, by staining the membrane with the probe after incubation of cells with 4 μM YydF^*^ for 5 and 20 min, respectively, we detected areas of dye accumulation (Figure 6B). Such lateral domains derive from local areas of altered lipid packing in the presence of YydF^*^. This result is in agreement with and further supports the YydF^*^-dependent membrane rigidification observed with laurdan.

## Discussion

In this work, we presented a comprehensive MOA analysis of the epipeptide (YydF^*^), a novel ribosomally-synthesized and post-translationally modified peptide (RiPP) (Benjdia et al., 2017a,b; Balty et al., 2019), produced by *B. subtilis*. We demonstrate that extrinsically added YydF^*^ targets the cell envelope and dissipates membrane potential via membrane permeabilization. These processes are accompanied by rapid loss of membrane fluidity, substantiated by lipid domain formation based in altered lipid packing. Disturbance of the lipid matrix causes drastic disorder of membrane associated proteins, thereby disrupting membrane function and homeostasis (Benjdia et al., 2017a,b; Balty et al., 2019). The global response of YydF^*^ upon *B. subtilis* treatment revealed a narrow and highly specific transcriptome profile. In addition to the upregulation of *liaIH*, a number of ECF-controlled stress genes and the *floT* operon were moderately induced, thereby supporting the determined effect of YydF^*^ on reducing membrane fluidity (Bach and Bramkamp, 2013). Daptomycin, another inducer of the Lia response (Wecke et al., 2009), shows some similarities to YydF^*^ regarding its MOA (Baltz et al., 2005; Müller et al., 2016; Scheinpflug et al., 2017b; Omardien et al., 2018). This antibiotic causes a comparable gradual membrane depolarization, but it lacks the membrane permeabilization observed for YydF^*^. Upon daptomycin treatment, cells undergo a drastic loss of membrane fluidity mediated by rearrangement of fluid lipid domains, which blocks cell wall synthesis, thereby ultimately causing cell death (Müller et al., 2016). As a consequence of both daptomycin and YydF^*^ treatment, *B. subtilis* encounters severe cell envelope stress and launches the LiaRS response, thereby inducing the *liaIH* operon to counteract daptomycin-/YydF^*^-dependent membrane perturbations (Wecke et al., 2009; Müller et al., 2016), and this study.

In agreement with the observed *liaIH* induction, our MIC experiments demonstrate that LiaIH—and not YydIJ—provides the primary resistance against the RiPP YydF^*^. Previous studies on LiaIH indicate that these co-localizing and directly interacting proteins are recruited to the site of damage perception to locally support membrane integrity (Wolf et al., 2010, 2012; Domínguez-Escobar et al., 2014). Interestingly, neither membrane depolarization nor the rigidification process *per se* are necessary for launching the LiaRS-dependent stress response in *B. subtilis*, since we observed none of these effects with the well-studied Lia-inducer bacitracin (Mascher et al., 2003; Radeck et al., 2016b). Bacitracin interferes with the lipid II cycle by blocking the recycle of the lipid carrier undecaprenyl pyrophosphate, which is essential for translocation of peptidoglycan precursors across the membrane (Storm and Strominger, 1973; Economou et al., 2013; Radeck et al., 2016b). The consistently opposite behavior—and therefore MOA—of bacitracin and YydF^*^ demonstrated in this work strongly suggests that a diverse range of mechanisms can trigger the LiaRS response. While a common feature of all stimuli inducing the Lia response is their interference with membrane-anchored steps of envelope homeostasis, the true nature of the signal perceived by the Lia system therefore still remains obscure, even after more than 15 years of research.

Next to YydF, *B. subtilis* produces at least two additional AMPs SKF and SDP, at the onset of stationary phase. These so-called cannibalism toxins are produced during starvation period and are postulated to delay full commitment to sporulation by killing sibling cells and feasting on their released nutrients (González-Pastor et al., 2003; Liu et al., 2010; González-Pastor, 2011). Interestingly, YydF and SDP show highly similar expression profiles (Benjdia et al., 2017b) and share some similarities in their MOA. The latter causes membrane potential dissipation and induces autolysis (Lamsa et al., 2012). Because of this mechanistic similarity and our previous observation that SDP and SKF also trigger a CESR in *B. subtilis* (Butcher et al., 2007; Höfler et al., 2016), it is tempting to postulate YydF as a third cannibalism-related AMP. However, the biological purpose of natively produced YydF still needs further investigations. YydF and the cannibalism toxins overlap in the mechanism of their production, as well as their ability to kill *B. subtilis* cells (Lamsa et al., 2012; Flühe et al., 2013; Höfler et al., 2016; Grell et al., 2018). However, SDP and SKF are under control of Spo0A, the master regulator governing sporulation (González-Pastor et al., 2003; Fujita et al., 2005), and no evidence so far points toward Spo0A influencing expression of the *yyd* operon. Instead, loss of ComA, the transcriptional regulator responsible for ultimately inducing competence for genetic transformation in *B. subtilis*, indirectly leads to a decrease of *yydFGHIJ* mRNA levels (Comella and Grossman, 2005). This observation points toward a potential involvement of YydF in competence development in *B. subtilis*. Therefore, it is tempting to suggest that YydF might be a fratricide similar toxin. The term fratricide has been coined for toxins produced by *Streptococcus pneumoniae* that enhance the diversity of genetic material during the competent state of this species (Steinmoen et al., 2002; Popp and Mascher, 2019). Future research will hopefully shed some light on the biological role that the YydF epipetide plays in the physiology of *B. subtilis* entering stationary phase.

### Experimental Procedures

#### Bacterial Strains and Growth Conditions

*Bacillus subtilis* and *E. coli* were grown at 37°C with aeration (at least 200 rpm agitation) in one of the following media: (i) Lysogeny broth (LB medium) or transformation medium (ii) MNGE [88.2% 1x MN medium (1.36% (w/v) dipotassium phosphate x 3 H_2_O, 0.6% (w/v) monopotassium phosphate, 0.1% (w/v) sodium citrate x H_2_O), 1.9% glucose, 0.19% potassium glutamate, 0.001% (w/v) ammonium ferric citrate, 0.005% (w/v) tryptophan, and 0.035% (w/v) magnesium sulfate]. For solid agar plates 1.5% (w/v) agar-agar was added. All used strains are listed in the supporting information Table S1. *E. coli* cells harboring a plasmid were selected using ampicillin (100 μg ml^−1^). *B. subtilis* cells carrying a resistance marker were selected using chloramphenicol (5 μg ml^−1^), spectinomycin (100 μg ml^−1^), or erythromycin combined with lincomycin (1 and 25 μg ml^−1^) for MLS. Transformation *of E. coli* and *B. subtilis* was performed as described previously (Harwood and Cutting, 1991)^1^.

#### DNA Manipulation

Plasmids were constructed using standard cloning techniques as described elsewhere (Sambrook and Russell, 2001). For DNA amplification via PCR, Q5® polymerase was used. Enzymes were purchased from New England Biolabs (NEB, Ipswich, MA, USA) and applied following their respective protocols. Positive *E. coli* clones were checked by colony PCR, using OneTaq® polymerase. All constructs were verified by sequencing. Successful integration of the plasmids into the *B. subtilis* genome were confirmed via colony PCR of respective up and down fragments. All primers and plasmids used in this study are listed in supporting Information Table S1.

#### Luciferase Assay

A detailed description of the procedure was described previously (Radeck et al., 2013; Popp et al., 2017). In brief, overnight cultures were grown in LB with respective antibiotics. Day cultures, without antibiotics, were inoculated 1:200 in fresh made pre-warmed medium as indicated and grown till and OD_600_ = 0.1−0.4. Subsequently, the cells were diluted to an OD_600_ = 0.05 or 0.01, for minimal and full media, respectively. Then, 100 μl of cells per sample were split in a 96-well-plate (black walls, clear bottom, Greiner Bio-One, Frickenhausen, Germany). If necessary, after 1 h, the cells were induced with active YydF (YydF^*^) peptide to the indicated final concentrations. The assay was performed using a Synergy™ NeoalphaB plate reader (BioTek, Winooski, VT, USA). The optical density as well as luminescence was measured every 5 min for at least 15 h.

#### Synthesis of YydF^*^

The epipeptide YydF^*^: WYFV^D^KSKENRWI^D^LGSGH (were “^D^” denotes D-amino acid residues) was synthesized using solid phase synthesis and standard procedures, as previously described (Benjdia et al., 2017b). Peptide purity was analyzed by reversed-phase HPLC (RP-HPLC) on an Agilent 1200 series infinity equipped with a LiChroCART RP-18e 5 μm column (Merck Millipore). YydF^*^ sequence was confirmed by high-resolution LC-MS/MS analysis (LTQ-Orbitrap mass spectrometer, Thermo Fisher Scientific).

#### RNA Sample Preparation and Sequencing

Overnight cultures of *B. subtilis* W168 (Nicolas et al., 2012) were prepared in LB (Sigma L3522) and incubated at 37°C, 220 rpm. On the next day, 10 ml day culture of LB (Sigma L3522) was inoculated 1:100 and grown till OD_600_ = 0.4. Subsequently, a second day culture of 200 ml LB (Sigma L3522) was started to an OD_600_ = 0.1. Once this second day culture reached OD_600_ = 0.5, cells were split into 25 ml aliquots and either exposed to 0.5 μM YydF^*^ (final concertation) or remained untreated for 10 min. After that, cells were directly placed onto ice and centrifuged at 4°C and 8,000 rpm for 3 min. The falcons containing the cell pellets were then frozen in liquid nitrogen and kept at −80°C. RNA isolation was performed with a phenol-chloroform extraction method. Briefly, cell pellets were resuspended in 200 μl killing buffer (20 mM Tris HCl pH 7.5, 5 mM MgCl_2_, 20 mM NaN_3_) and immediately disrupted for 2 min at 2,600 rpm using a micro-dismembrator with a homogenizer vessel and a steel ball that have been snap frozen in liquid nitrogen. The cell powder was resuspended in 4 ml pre-warmed lysis buffer (4 M GTC, 0,025 M NaOAc pH 5.2, 0.5% lauroylsarcosine). One volume phenol-chloroform-isoamyl alcohol 25:24:1 pH 5.5 was added. After centrifugation for 5 min at 12,000 rcf, the supernatant was mixed with chloroform-isoamyl alcohol (24:1, pH 8). After centrifugation, a NaOAc/isopropanol precipitation was performed and RNA pellets were washed twice with 70% ethanol. Total RNA was resuspended in DEPC-treated water. rRNA was subtracted from the samples with the Illumina Ribo-Zero rRNA removal Kit (Bacteria). The cDNA library was prepared using the NEB Ultra RNA directional prep kit for Illumina and sequencing was performed on an Illumina HiSeq3000 system. Sequencing reads were mapped to the BaSysBio 168 strain (NC_000964.3) using Bowtie2 (Langmead and Salzberg, 2012). The software program featureCounts of the Subread package (Liao et al., 2014) was applied to generate counts for known genes. Differentially expressed genes were identified using the R/Bioconductor package DESeq2 (Love et al., 2014). The raw and processed RNA sequencing data obtained in this study has been deposited at the NCBIs Gene Expression Omnibus (Edgar et al., 2002) and is accessible via the GEO accession number GSE140605.

#### Minimum Inhibitory Concertation (MIC) Assay

The tested strains were grown overnight in LB supplemented with the necessary antibiotics for selection. From these overnight cultures, cells were diluted to a final OD_600_ = 0.01 in fresh pre-warmed LB. The samples were then split in a 96-well-plate (black walls, clear bottom, Greiner Bio-One, Frickenhausen, Germany) and directly exposed to the YydF^*^ AMP at the depicted concentration and incubated for 24 h. Endpoint measurement of the optical density was plotted against the tested concentrations. The lowest AMP concentration at which each strain showed an OD_600_ = 0.01 determined the minimum inhibitory concentration.

#### Membrane Depolarization Assays

To investigate the MOA of the synthesized YydF^*^ membrane depolarization assays using the voltage sensitive Disc_3_(5) (Anaspec) were performed. These assay are well-described elsewhere (Te Winkel et al., 2016). In brief, to perform the spectroscopic assay, *B. subtilis* wild type was grown in LB overnight followed by the cultivation in a fresh LB day culture. Cells were grown till exponential phase, then diluted to a final OD = 0.2 in LB supplemented with BSA (0.5 mg ml^−1^). Cells were transferred into a 96-well-plate (black walls, clear bottom, Greiner Bio-One, Frickenhausen, Germany) and the auto fluorescence of *B. sutbilis* was recorded at 610 nm (excitation) and 660 nm (emission) for 3 min using a Synergy™ NeoalphaB plate reader (BioTek, Winooski, VT, USA). Subsequently, 1 μM Disc_3_(5) was added and the in-cooperation of the dye into the membrane was monitored for another 7 min till steady fluorescence levels were reached. The antibiotics of interested were added to the concentrations as indicated and the changes of fluorescence were obtained for an additional hour at 1 min intervals.

To further characterize the effects of observed depolarization, the membrane dye Disc_3_(5) was combined with Sytox green to perform fluorescence microscopy (Kepplinger et al., 2018). For that, *B. subtilis* was grown as described above. Cells were again diluted to an OD = 0.2 in fresh LB supplemented with BSA (0.5 mg ml^−1^). Subsequently, 200 μl were transferred to a 2 ml reaction tube. and mixed with 1 μM Disc_3_(5) and 50 nm Sytox green (Thermo) final concentration. The reaction tube was incubated in an Eppendorf Thermomixer with opened lid, to allow sufficient aeriation, at 37°C and 1,000 rpm for 5 min. Addition of the antibiotics for 10 min was carried out right after and then 2 μl of cells were transferred to an agarose pad (1% Ultra-pure Agarose, Invitrogen). Fluorescence microscopy was performed using an Axio Observer 7 inverse microscope (Carl Zeiss, Jena, Germany) equipped with standard Cye5 (Ex: 650/EM: 673) and eGFP (Ex: 488/EM 509) filter sets.

The acquired microscope images were analyzed using the open source platform Fiji (Schindelin et al., 2012) equipped with the plugin MicrobeJ (Ducret et al., 2016). For each condition tested, at least 300 cells were marked as region of interest either by the plugin or manually. To quantify the effects of depolarization and membrane permeabilization mean pixel intensities of both channels (Cye5 and eGFP) were obtained.

#### Membrane Fluidity Assay

To measure altering membrane fluidity due to active YydF^*^ treatment, Laurdan generalized polarization (GP) assay was performed as described previously (Scheinpflug et al., 2017a). For this, *B. subtilis* wild type cells were incubated overnight in LB, refreshed in a day culture supplemented with 0.1% glucose and grown till exponential phase. Next, cells were diluted to OD = 0.2 and incubated with 10 μM Laurdan for 5 min under shacking conditions with aeriation. Cells were then washed four times with PBS (supplemented with 0.1% glucose) and the fourth supernatant was collected to serve as negative control without cells. Subsequently, pelleted cells were resuspended and transferred into a 96-well-plate (black walls, clear bottom, Greiner Bio-One, Frickenhausen, Germany). Fluorescence was measured at excitation of 350 nm and the emission was recorded at 435 nm as well as 500 nm using a Synergy™ NeoalphaB plate reader (BioTek, Winooski, VT, USA). After 5 min, compounds were added to the final concentrations as indicated and the fluorescence measurement was continued for an additional hour. Laurdan GP values of each time point were calculated according to the formula described in Scheinpflug et al. (2017a).

#### Nile Red Fatty Acid Packing Assay

To investigate lipid domain formation upon YydF^*^ treatment, cells were stained with the fluorescent dye nile red as described previously (Scheinpflug et al., 2017b). In brief, cells were grown to exponential phase and then diluted to an optical density of 0.2. After that, cells were exposed to 4 μM YydF^*^ and incubated for 5 or 20 min, respectively. Immediately prior performing microscopy cells were stained with nile red (final concentration 1 μg ml^−1^). Fluorescence microscopy was carried out using an Axio Observer 7 inverse microscope (Carl Zeiss, Jena, Germany).

#### Data Analysis and Statistical Procedures

Growth and luminescence measurements were performed in biological duplicates and technical triplicates. From the values obtained for each time point, mean values and standard deviation (±) were calculated and plotted (Figure 2). The data derived from the RNA-seq experiments were obtained in biological and technical triplicates. Genes were considered relevant that differed at least four-fold relative to the non-induced reference samples with an adjusted *p*-value below 0.05. No genes where down-regulated upon 0.5 μM YydF^*^ treatment (Figure 3, Table 1 and Table S2). MIC experiments were performed at least in biological and technical duplicates and mean values as well as standard deviation (±) are displayed (Figure 4). For the depolarization assays performed in the plate reader, each condition (antimicrobial compound) was tested in biological duplicates and technical triplicates. From the raw data, mean values and standard deviation (±) were calculated and plotted as function of time (Figure 5A). Depolarization and Sytox Green assays performed microscopically were evaluated using Fiji and the plugin MicrobeJ (Schindelin et al., 2012; Ducret et al., 2016). Here, pictures obtained from biological and technical triplicates were analyzed and regions of interest (i.e., cells) were chosen by the algorithm and manually corrected if necessary. Finally, from each condition tested, at least 300 cells were included in the final dataset and mean pixel intensities were plotted (Figures 5B,C). Data obtained from the fluidity assays (Figure 6A) were performed in biological and technical triplicates. Emission spectra of Laurdan were obtained once every minute and mean values as well as standard deviation (±) were calculated and plotted as a function of time. Microscopy of *B. subtilis* cells stained with Nile Red (Figure 6B) was performed in biological and technical duplicates and full-size pictures are provided as Figures S3, S4, respectively.

## Data Availability Statement

The raw data supporting the conclusions of this article will be made available by the authors, without undue reservation, to any qualified researcher.

## Author Contributions

PP and TM planed the project and wrote the manuscript. PP conducted the experiments. PP, TM, AB, HS, and OB analyzed and interpreted the data.

### Conflict of Interest

The authors declare that the research was conducted in the absence of any commercial or financial relationships that could be construed as a potential conflict of interest.

## Footnotes

^1^http://www.openwetware.org/wiki/Transforming_chemically_competent_cells; http://openwetware.org/wiki TOP chemically competent cells (accessed February 19, 2017).

## Supplementary Material

Figure S1 Growth curves and luciferase activity of *B. subtilis* P_*liaI*_-*lux* strains in full medium. Upper panel depict growth curves, lower panel show luminescence values normalized over optical density. Measurements were taken every 5 min over a time period of 15 h. Growth in full medium of P_*liaI*_-*lux* strains in the wild type background and individual *yyd* mutants.

Figure S2 Growth curves of *B. subtilis* wild type in full medium. Growth in full media of *B. subtilis* wild type, challenged with various concentrations of YydF^*^ after 1 h incubation. Measurements were taken every 5 min over a time period of 15 h.

Figure S3 Effect of YydF^*^ on *B. subtilis* membranes lipid packing state. Overlay of phase contrast and fluorescence images of *B. subtilis* cells stained with the membrane dye nile red after exposure to 4 μM YydF^*^ for 5 min.

Figure S4 Effect of YydF^*^ on *B. subtilis* membranes lipid packing state. Overlay of phase contrast and fluorescence images of *B. subtilis* cells stained with the membrane dye nile red after exposure to 4 μM YydF^*^ for 20 min.

Table S1 Strains, primers and plasmids.

Table S2 Complete RNA-sequencing (RNA-seq) profile of *B. subtilis* upon YydF^*^ treatment.
