# Supplementary figures and images for: The Epipeptide YydF Intrinsically Triggers the Cell Envelope Stress Response of Bacillus subtilis and Causes Severe Membrane Perturbations
Source: Front Microbiol. 2020 Feb 11;11:151. doi: 10.3389/fmicb.2020.00151 (PMC7026026; doi:10.3389/fmicb.2020.00151)

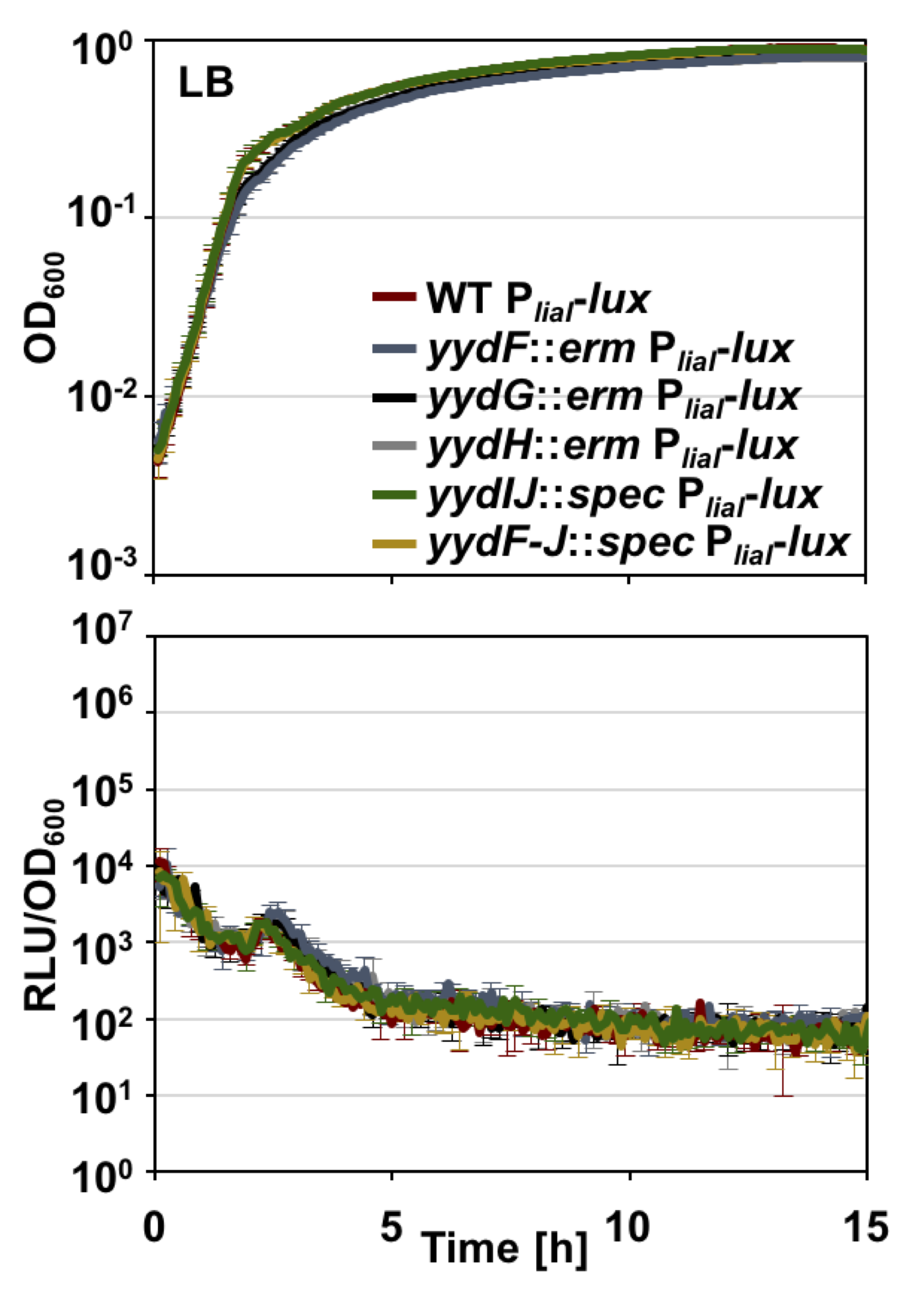

Supplement: Figure S1 — Growth curves and luciferase activity of B. subtilis PliaI-lux strains in full medium. Upper panel depict growth curves, lower panel show luminescence values normalized over optical density. Measurements were taken every 5 min over a time period of 15 h. Growth in full medium of PliaI-lux strains in the wild type background and individual yyd mutants. [file Image_1.TIFF]

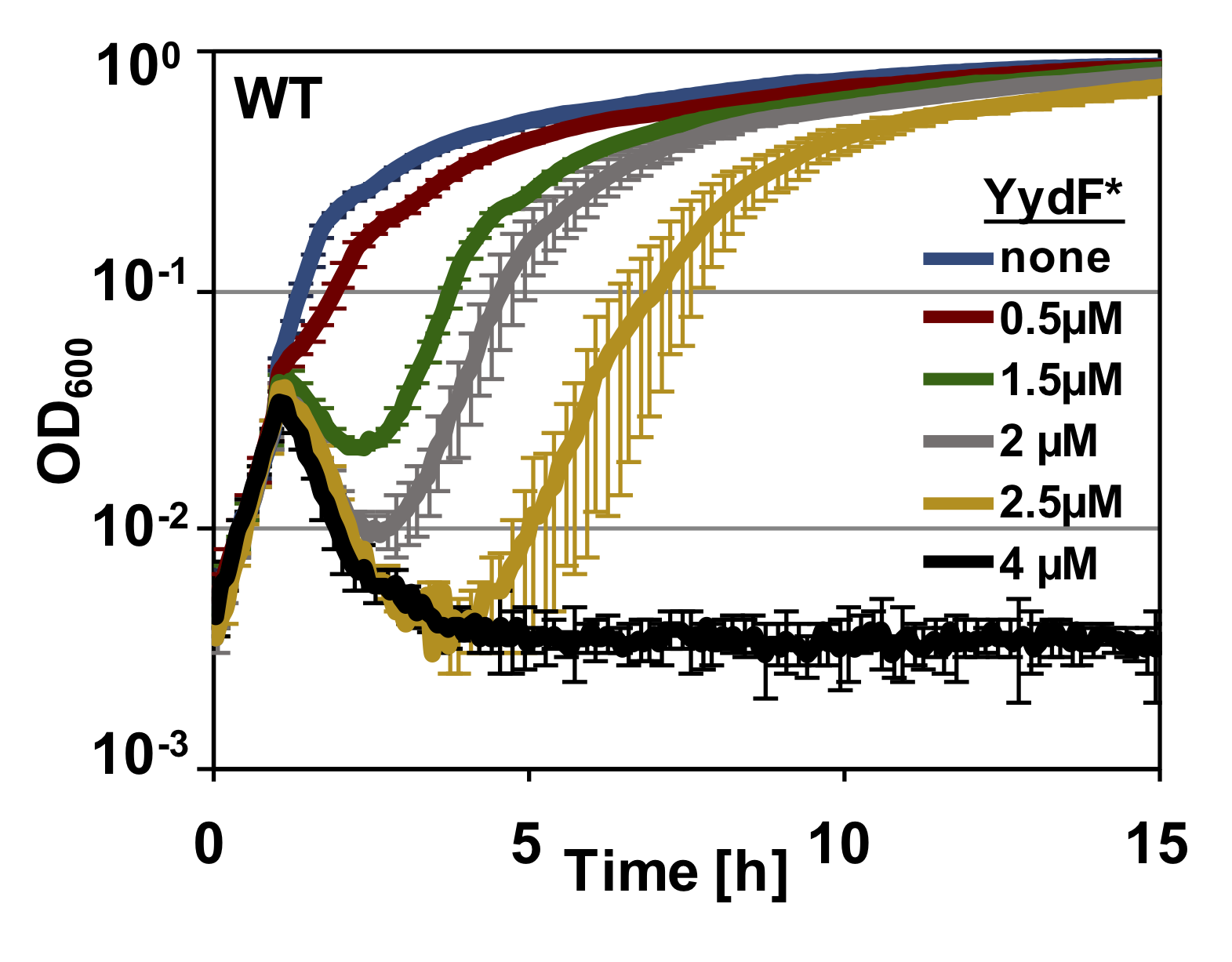

Supplement: Figure S2 — Growth curves of B. subtilis wild type in full medium. Growth in full media of B. subtilis wild type, challenged with various concentrations of YydF* after 1 h incubation. Measurements were taken every 5 min over a time period of 15 h. [file Image_2.TIFF]

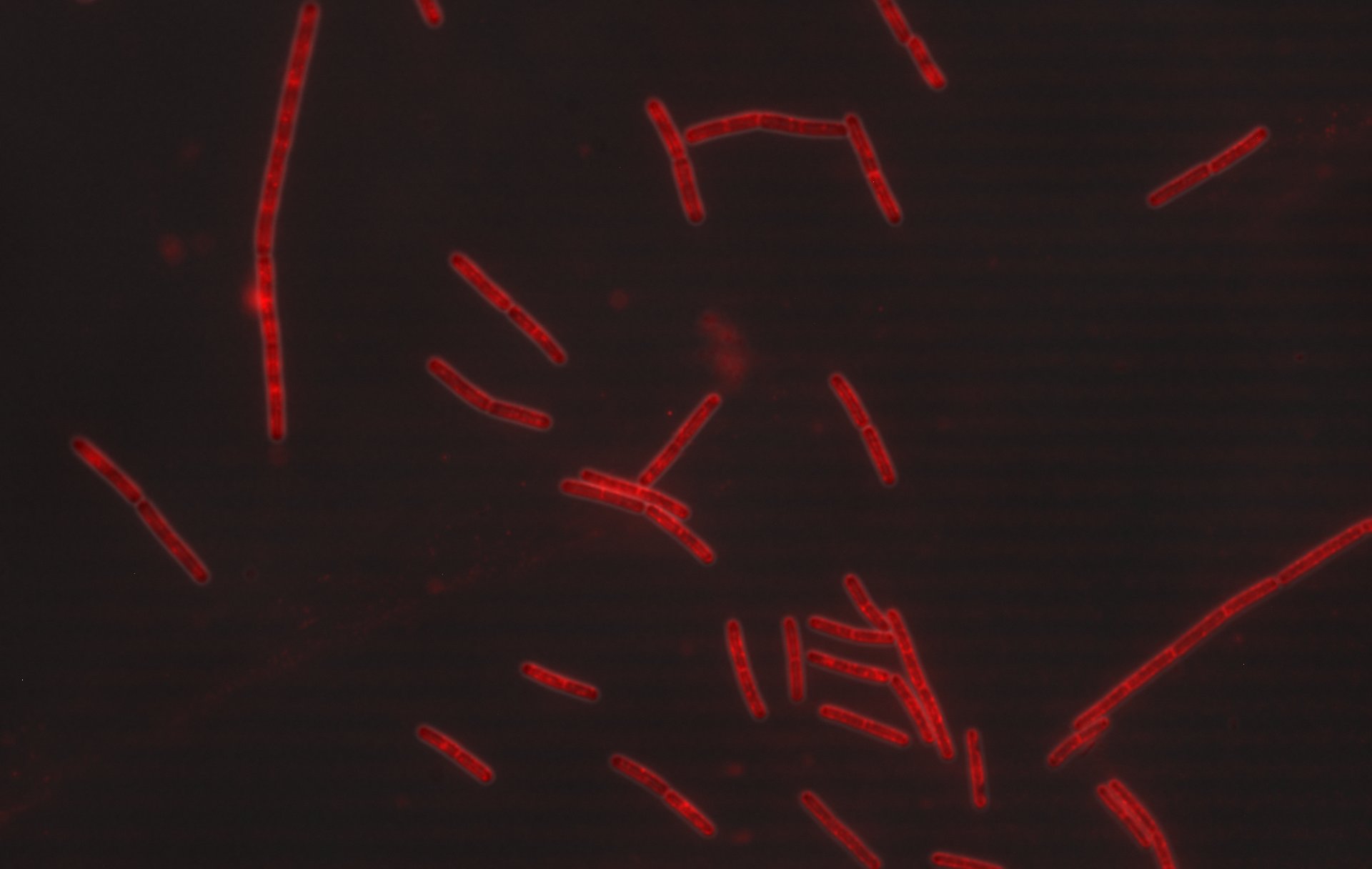

Supplement: Figure S3 — Effect of YydF* on B. subtilis membranes lipid packing state. Overlay of phase contrast and fluorescence images of B. subtilis cells stained with the membrane dye nile red after exposure to 4 μM YydF* for 5 min. [file Image_3.JPEG]

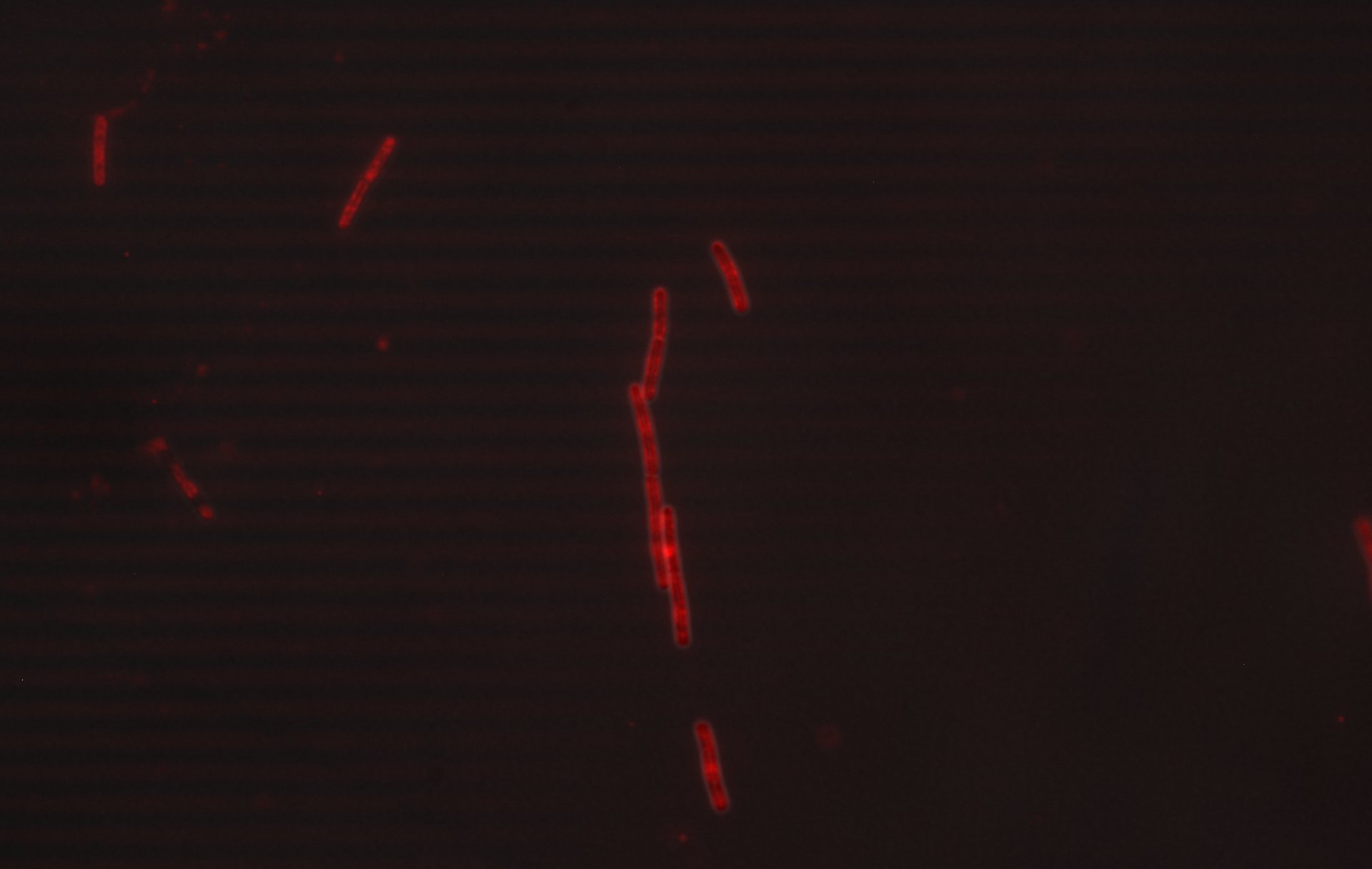

Supplement: Figure S4 — Effect of YydF* on B. subtilis membranes lipid packing state. Overlay of phase contrast and fluorescence images of B. subtilis cells stained with the membrane dye nile red after exposure to 4 μM YydF* for 20 min. [file Image_4.JPEG]
