# Supplementary material for: The Epipeptide YydF Intrinsically Triggers the Cell Envelope Stress Response of Bacillus subtilis and Causes Severe Membrane Perturbations
Source: Front Microbiol. 2020 Feb 11;11:151. doi: 10.3389/fmicb.2020.00151 (PMC7026026; doi:10.3389/fmicb.2020.00151)
Supplement: Table S1 — Strains, primers and plasmids. [file Table_1.docx]

Table S1: Strains, Primers and Plasmids

| **Name** | **Description and comment** | **Source** |
| --- | --- | --- |
| *Bacillus subtilis* W168 | wild type, *trpC2* | lab strain |
| *Bacillus subtilis* W168 | wild type, *trp^+^* | (Nicolas et al., 2012) |
| TMB1732 | W168 *yydF-J*::*spec* | This study, (Butcher et al., 2007) |
| TMB1733 | W168 *yydIJ*::*spec* | This study, (Butcher et al., 2007) |
| TMB3822 | W168 *sacA*::P*_liaI_*-*luxABCDE* | (Popp et al., 2017) |
| TMB4265 | W168 *yydF*::*erm* *sacA*::P*_liaI_*-*luxABCDE* | This study, (Koo et al., 2017) |
| TMB4264 | W168 *yydG*::*erm* *sacA*::P*_liaI_*-*luxABCDE* | This study, (Koo et al., 2017) |
| TMB4263 | W168 *yydH*::*erm sacA*::P*_liaI_*-*luxABCDE* | This study, (Koo et al., 2017) |
| TMB3834 | W168 *yydIJ*::*spec* *sacA*::P*_liaI_*-*luxABCDE* | This study |
| TMB3828 | W168 *yydF-J*::*spec* *sacA*::P*_liaI_*-*luxABCDE* | This study |
| TMB2841 | W168 *sacA*::*luxABCDE (empty vector)* | (Pinto et al., 2018) |
| TMB1151 | W168 ∆*liaIH* | (Toymentseva et al., 2012) |
| TMB4188 | W168 *yydIJ*::*spec* ∆*liaIH* | This study |
| **Name** | **Sequence 5'-3'** | **Source / Name** |
| TM2895 | GATCGAATTCGCGGCCGCTTCTAGAGATTGGCCAAAGCAGAAAGGTCC | P*_liaI_* fwd |
| TM2896 | GATCACTAGTATCGTTTTCCTTGTCTTCATCTTATAC | P*_liaI_* rev |
| TM2262 | GAGCGTAGCGAAAAATCC | pBS3C*lux*checkfwd |
| TM2263 | GAAATGATGCTCCAGTAACC | pBS3C*lux*checkrev |
| TM2505 | CTGATTGGCATGGCGATTGC | pBS3C*lux* *sacA* front check fwd |
| TM2506 | ACAGCTCCAGATCCTCTACG | pBS3C*lux* *sacA* front check rev |
| TM2507 | GTCGCTACCATTACCAGTTG | pBS3C*lux* *sacA* back check fwd |
| TM2508 | TCCAAACATTCCGGTGTTATC | pBS3C*lux* *sacA* back check rev |
| TM0716 | CAGGAAAAGGCCATTTTACC | *ery*-check-fwd |
| TM0717 | AAATCGTCAATTCCTGCATGT | *ery*-check-rev |
| TM2759 | GAGGGGCTAGAGGACTATAGG | *yydF* check fwd |
| TM0149 | CGTATGTATTCAAATATATCCTCCTCAC | *spec*-check rev |
| TM5729 | gatcGGAATTCGCGGCCGCTTCTAGAG TAGCAGCGCCAACTGATATCATTG | P*_yydFa550-205_* fwd |
| **Name** | **Description** | **Source** |
| pBS3C*lux* | *sacA*´…´*sacA, luxABCDE, cat, bla* | (Radeck et al., 2013) |

Adebali, O., Ortega, D. R., and Zhulin, I. B. (2015). CDvist: a webserver for identification and visualization of conserved domains in protein sequences. Bioinformatics 31, 1475–1477. doi:10.1093/bioinformatics/btu836.

Koo, B.-M., Kritikos, G., Farelli, J. D., Todor, H., Tong, K., Kimsey, H., et al. (2017). Construction and Analysis of Two Genome-Scale Deletion Libraries for Bacillus subtilis. Cell Syst 4, 291–305.e7. doi:10.1016/j.cels.2016.12.013.

Pinto, D., Vecchione, S., Wu, H., Mauri, M., Mascher, T., and Fritz, G. (2018). Engineering orthogonal synthetic timer circuits based on extracytoplasmic function σ factors. Nucleic Acids Res. 46, 7450–7464. doi:10.1093/nar/gky614.

Toymentseva, A. A., Schrecke, K., Sharipova, M. R., and Mascher, T. (2012). The LIKE system, a novel protein expression toolbox for Bacillus subtilis based on the liaI promoter. Microb. Cell Fact. 11, 143. doi:10.1186/1475-2859-11-143.
