# Supplementary material for: The Epipeptide YydF Intrinsically Triggers the Cell Envelope Stress Response of Bacillus subtilis and Causes Severe Membrane Perturbations
Source: Front Microbiol. 2020 Feb 11;11:151. doi: 10.3389/fmicb.2020.00151 (PMC7026026; doi:10.3389/fmicb.2020.00151)
Supplement: Table S2 — Complete RNA-sequencing (RNA-seq) profile of B. subtilis upon YydF* treatment. [file Table_2.docx]

Table S2: Complete RNA-sequencing (RNA-seq) profile of *B. subtilis* upon YydF* treatment

| **Gene** | **Operon^1^** | **Regulation/ Regulon^1^** | **log2 fold change** | **p-value** | **Protein Domains^2^** | **Description^1^** |
| --- | --- | --- | --- | --- | --- | --- |
| *liaH* | ***liaIH*** | LiaRS | -4.3 | 3.2·10^-189^ | PspA_IM30 | phage-shock protein homolog; resistance against envelope stress |
| *liaI* |  |  | -3.9 | 8.5·10^-95^ | DUF5362 | transmembrane protein; membrane anchor of LiaH |
| *hisG* | ***hisZGDBHAFI*** | YlxR | -3.7 | 5.6·10^-55^ | HisG | ATP phosphoribosyltransferase |
| *hisD* |  |  | -3.6 | 1.6·10^-83^ | Histidinol_dh | histidinol dehydrogenase |
| *hisB* |  |  | -3.5 | 3.4·10^-60^ | IGPD | imidazoleglycerol-phosphate dehydratase |
| *hisH* |  |  | -3.5 | 1.7·10^-68^ | GATase | imidazole glycerol phosphate synthase; glutaminase subunit |
| *hisF* |  |  | -3.4 | 6.0·10^-73^ | His_biosynth | imidazole glycerol phosphate synthase; synthase subunit |
| *hisI* |  |  | -3.3 | 3.1·10^-71^ | PRA-CH / PRA-PH | phosphoribosyl-AMP cyclohydrolase / phosphoribosyl-ATP pyrophosphohydrolase |
| *hisA* |  |  | -3.2 | 5.9·10^-68^ | His_biosynth | phosphoribosylformimino-5-aminoimidazole carboxamide ribotide isomerase |
| *hisZ* |  |  | -3.1 | 2.4·10^-52^ | tRNA-synt_His | histidyl-tRNA synthetase |
| *floT* | ***yuaF-floT-yuaI*** | SigW | -2.3 | 5.6·10^-53^ | Band_7 / Flot | membrane-associated scaffold protein involved in controling membrane fluidity |
| *yuaF* |  |  | -2.2 | 1.3·10^-17^ | NfeD | role in maintaining membrane integrity during conditions of cellular stress |
| *yuaI* |  |  | -2.1 | 2.6·10^-27^ | Acetyltransf_1 | n/a |
| *bcaP* | none | CodY | -3.3 | 2.3·10^-49^ | AA_permease_2 / AA_permease_C | branched-chain amino acid transporter |
| *ilvD* | none | CodY | -2.7 | 1.6·10^-81^ | ILVD_EDD | dihydroxy-acid dehydratase |
| *yrhH* | ***yrhH****-fatR-yrhJ* | SigW, SigM, SigX, SigV | -2.7 | 3.7·10^-69^ | Methyltransf_11 | similar to methyltransferase |
| *hpf* | *yvzC-fliD-filS-fliT-****hpf*** | PhoP, SigB, SigH, SigD | -2.4 | 5.9·10^-78^ | Ribosomal_S30AE / Ribosomal_S30AE_C | general stress protein |
| *iseA* | none | WalR | -2.2 | 2.1·10^-35^ | Endopep_inhib | inhibits in vitro activity of cell wall endopeptidases, inhibits cell separation |
| *yqjL* | none | SigW, SigB, SigM, SigV | -2.2 | 6.9·10^-91^ | Abhydrolase_1 / Ndr | general stress protein |
| *sasA* | none | SigW, SigM, SigV | -2.2 | 2.5·10^-77^ | RelA_SpoT | (p)ppGpp synthetase, small alarmone synthetase |
| *nhaX* | none | SigB, NhaX | -2.1 | 8.7·10^-40^ | Usp | general stress protein |

^1^(Zhu and Stülke, 2018), ^2^(Adebali et al., 2015)
